# Reversible Central Sleep Apnea in Acute Necrotizing Encephalopathy of Childhood: A Case Report

**DOI:** 10.7759/cureus.110081

**Published:** 2026-06-01

**Authors:** Badar Al Dhouyani, Amal R Al-Naimi

**Affiliations:** 1 Pediatric Pulmonology, Sidra Medicine, Doha, QAT

**Keywords:** acute necrotizing encephalopathy, anec, bipap, brainstem dysfunction, central sleep apnea, noninvasive ventilation, pediatric neurology, polysomnography, reversible respiratory failure, sleep-disordered breathing

## Abstract

Acute necrotizing encephalopathy of childhood (ANEC) is a rare, rapidly progressive neuroinflammatory disorder typically triggered by viral infections and characterized by bilateral thalamic and brainstem involvement. While its neurological manifestations are well described, its impact on sleep-related respiratory control remains underrecognized. Brainstem dysfunction in ANEC may impair central ventilatory drive, resulting in central sleep apnea.

We report a previously healthy five-year-old boy who presented with fever, headache, and prolonged generalized seizure, followed by decreased consciousness. Neuroimaging revealed extensive bilateral involvement of the thalami, brainstem, and cerebellum, consistent with ANEC. During recovery, he developed severe central sleep apnea, with polysomnography demonstrating an apnea-hypopnea index (AHI) of 102 events per hour, entirely central in nature. Non-invasive ventilation using bi-level positive airway pressure (BiPAP) led to complete resolution of respiratory events. Despite persistent neurological impairment, follow-up polysomnography after one year showed marked improvement, with only mild residual central events (central apnea index (CAI) 3.4 events/hour) and no clinically significant hypoventilation, allowing discontinuation of ventilatory support.

This case highlights central sleep apnea as a potentially severe but reversible complication of ANEC, likely related to transient brainstem dysfunction. Early recognition and evaluation with polysomnography are essential, particularly in patients with brainstem involvement. Timely initiation of non-invasive ventilation may stabilize respiratory function and allow eventual weaning as central control improves. This report expands the clinical spectrum of ANEC and underscores the importance of respiratory monitoring in affected children.

## Introduction

Acute necrotizing encephalopathy of childhood (ANEC) is a rare neuroinflammatory condition marked by rapid neurological decline following a febrile illness. It typically involves the thalami and brainstem. While its neurological manifestations are well documented, the effects of ANEC on sleep and respiratory regulation remain underrecognized [1].

Respiratory control during sleep is primarily governed by the brainstem. Damage to these regions, as occurs in ANEC, may impair central ventilatory drive, leading to central sleep apnea or hypoventilation [2].

We describe a child with ANEC who developed severe central sleep apnea requiring nocturnal respiratory support. However, after one year, repeat polysomnography showed complete resolution of respiratory events, and respiratory support was no longer necessary, despite incomplete neurological recovery.

## Case presentation

A previously healthy five-year-old boy presented with a one-day history of fever and headache, followed by a generalized tonic-clonic seizure lasting approximately 15 minutes. He was transported by EMS and treated with intravenous midazolam. On arrival, he had a decreased level of consciousness and high-grade fever. Initial vital signs were as follows: heart rate 110 beats per minute, blood pressure 100/60 mmHg, respiratory rate 26 breaths per minute, oxygen saturation 100% on room air, and end-tidal carbon dioxide (EtCO_2_) 36 mmHg. Neurological examination revealed poor responsiveness, pinpoint pupils, increased tone in the right lower limb, and signs of brainstem dysfunction, including impaired upward gaze and an absent doll’s eye reflex.

Admission laboratory results were unremarkable, as shown in Table 1. Viral testing was positive for parvovirus B19 and parainfluenza virus. Neuroimaging demonstrated extensive bilateral involvement of the thalami, deep gray nuclei, brainstem, mesial temporal regions, and cerebellum, with hemorrhagic and necrotic changes consistent with ANEC (Figure 1). Electroencephalography (EEG) showed diffuse slowing without epileptiform discharges (Figure 2).

**Table 1:** Laboratory results on admission. Laboratory results for our patient with the normal range. AST: aspartate aminotransferase; ALT: alanine aminotransferase

Test	Patient result	Unit	Reference range
Sodium (Na)	136	mmol/L	135-145
Potassium (K)	3.4	mmol/L	3.5-5.0
Chloride (Cl)	106	mmol/L	98-107
Blood urea nitrogen (BUN)	2.8	mmol/L	2.5-7.1
Creatinine	16	µmol/L	20-60 (child)
Glucose (random)	5.4	mmol/L	3.9-7.8
Calcium (total)	2.24	mmol/L	2.1-2.6
Lactate	1.4	mmol/L	0.5-2.2
AST	34	U/L	<40
ALT	17	U/L	<40
Albumin	36	g/L	35-50
Total bilirubin	6	µmol/L	0-21

**Figure 1:**
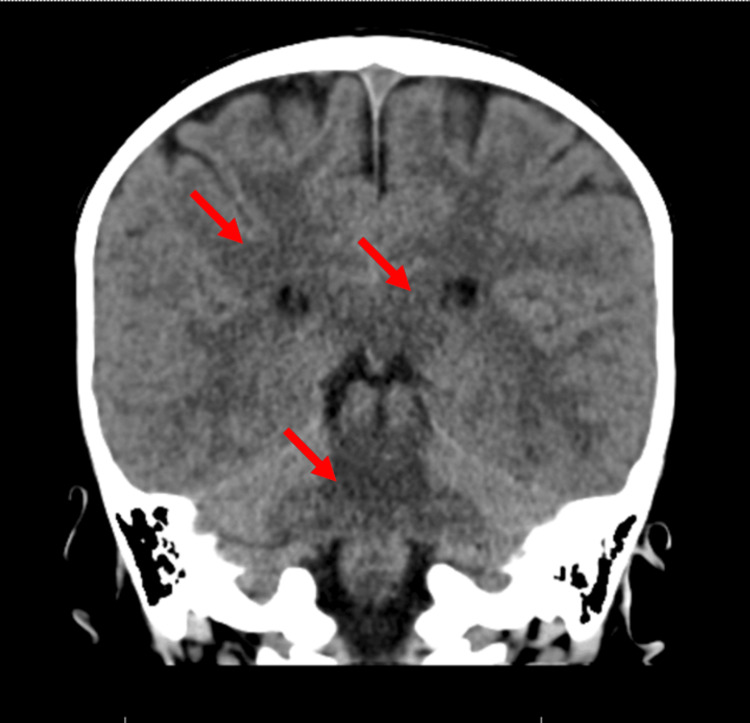
Non-contrast computed tomography (CT) of the brain. Coronal non-contrast CT brain image demonstrating symmetric hypoattenuation involving the bilateral thalami (red arrows), with associated diffuse cerebral edema and loss of normal gray-white matter differentiation. These findings are characteristic of acute necrotizing encephalopathy of childhood (ANEC) in the appropriate clinical context.

**Figure 2:**
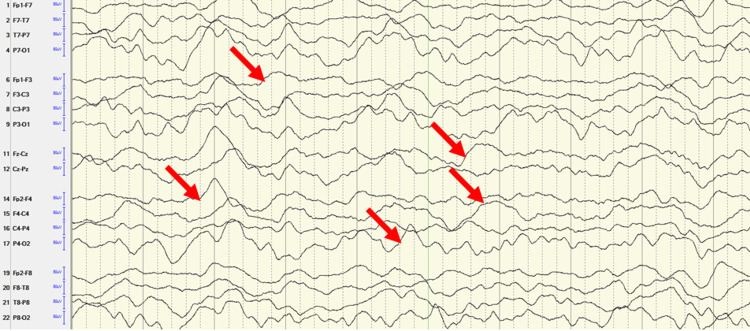
Electroencephalography (EEG) demonstrating generalized slow wave. Longitudinal bipolar montage EEG showing diffuse high-amplitude polymorphic delta slowing (red arrows), predominantly over the bilateral frontotemporal and posterior regions without a clear posterior dominant rhythm. No definite epileptiform discharges or electrographic seizures are identified in this segment. These findings are consistent with moderate-to-severe diffuse cerebral dysfunction/encephalopathy.

The patient required admission to the pediatric intensive care unit, where he was intubated for two weeks. Treatment included ceftriaxone, acyclovir, levetiracetam, pulse methylprednisolone (30 mg/kg), plasma exchange, and tocilizumab. He was subsequently extubated but remained minimally conscious, with spasticity requiring baclofen and gastrostomy feeding.

During recovery, he developed central sleep apnea with bradypnea. Initial polysomnography revealed severe central sleep apnea, with an apnea-hypopnea index (AHI) of 102 events per hour (all central events), an average oxygen saturation of 97% with a nadir of 89%, and transcutaneous CO₂ up to 39.6 mmHg (Figure 3).

**Figure 3:**
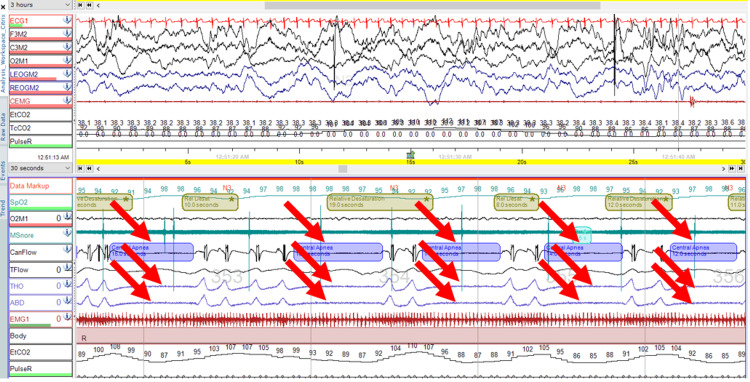
Polysomnography on admission. Representative 30 s polysomnography epoch demonstrating recurrent central sleep apnea events (red arrows). The airflow signal (CanFlow) shows cessation of airflow lasting approximately 10-15 s, accompanied by absence of thoracoabdominal effort (THO, ABD), consistent with central apnea. These events are associated with intermittent oxygen desaturations (SpO₂).

Bi-level positive airway pressure (BiPAP) therapy was initiated and titrated to 10/4 cmH₂O with a backup rate of 20 breaths per minute, resulting in complete resolution of respiratory events. Follow-up polysomnography after one year demonstrated significant improvement, with a marked reduction in central events. A subsequent diagnostic sleep study showed an AHI of 7.7 events per hour, obstructive AHI (OAHI) of 0 events per hour, central AHI (CAHI) of 7.6 events per hour, and central apnea index (CAI) of 3.4 events per hour.

Average oxygen saturation was 97%, with a nadir of 93%, and there was no significant hypoventilation with average EtCO_2_ 45 mmHg, peak 56 mmHg, with only 1.9% of total sleep time with EtCO_2_ above 50 mmHg, which is insignificant (Figure 4). No clinically significant respiratory events were identified, and discontinuation of non-invasive ventilation was recommended.

**Figure 4:**
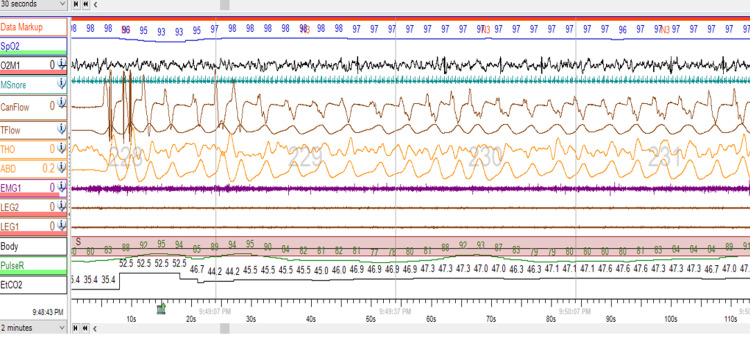
Polysomnography performed one year later. The figure demonstrates stable breathing with preserved airflow and synchronous thoracoabdominal movements, indicating resolved central sleep apnea. The airflow signal (CanFlow) shows regular, continuous airflow. Thoracoabdominal channels (THO, ABD) demonstrate synchronous respiratory effort without evidence of paradoxical movement. Oxygen saturation (SpO₂) remains stable without significant desaturation events. End-tidal carbon dioxide (EtCO₂) demonstrates normal values without abrupt fluctuations. These findings represent normal respiratory control during sleep.

## Discussion

This case highlights ANEC as a clinically relevant cause of central sleep-disordered breathing in children-an association that remains underrecognized in the literature. Seo et al. reported the outcomes of ANE in six children with an age range of 1-7 years. Only 12.8% of cases had full recovery; mild-to-moderate sequelae were reported in 23%, severe sequelae in 33.3%, and death in 28.2% [3]. In contrast, our patient demonstrated poor neurological recovery but marked improvement in central sleep apnea. This divergence suggests a potentially reversible mechanism affecting respiratory control despite persistent neurological deficits.

In this patient, the combination of bilateral thalamic and brainstem involvement on neuroimaging, along with clinical signs of brainstem dysfunction, provides a strong anatomical basis for disruption of sleep-related respiratory control. This was reflected on polysomnography by severe central sleep apnea (AHI 102 events/hour, entirely central), indicating profound instability of automatic ventilatory drive during sleep. From a physiological perspective, respiratory control during sleep-particularly in non-rapid eye movement (NREM) stages-relies predominantly on brainstem autonomic networks, with reduced cortical compensation [4].

Damage to these structures, as seen in ANEC, may preferentially manifest during sleep as central apnea or hypoventilation, even when daytime respiratory function appears relatively preserved. The complete resolution of respiratory events with BiPAP therapy (AHI 0), followed by progressive improvement and near normalization on follow-up polysomnography, supports a reversible dysfunction of central respiratory control rather than permanent structural failure. This likely reflects a transient neurophysiological consequence of inflammatory brainstem injury following viral-associated ANEC [5].

This case expands the clinical spectrum of ANEC to include sleep-related breathing disorders and underscores the importance of early sleep evaluation and longitudinal monitoring. Recognizing this association is essential, as timely initiation of non-invasive ventilation can stabilize nocturnal gas exchange and help prevent complications related to untreated sleep-disordered breathing. This case highlights ANEC as a severe neuroinflammatory condition with important implications for sleep regulation and respiratory control-areas that remain underrecognized in the pediatric literature.

Consistent with previously reported pediatric cases, our patient presented with rapid neurological deterioration following a febrile illness, in keeping with the well-established infection-triggered hyperinflammatory response underlying ANEC [1,2]. The pathophysiology is thought to involve cytokine-mediated injury rather than direct viral invasion, leading to disruption of the blood-brain barrier and preferential involvement of deep gray matter structures, particularly the thalami and brainstem [6]. This concept was first described in early pediatric cohorts, in which ANEC was characterized as an infection-associated encephalopathy driven by systemic cytokine dysregulation [6,7]. These regions play a critical role in sleep-wake regulation, arousal, and autonomic respiratory control, providing a biological basis for the association between ANEC and sleep-related disturbances in children.

Pediatric studies consistently identify bilateral thalamic involvement as the hallmark neuroimaging feature of ANEC, often accompanied by lesions in the brainstem, cerebellum, and white matter, which are associated with worse outcomes [7]. Larger series have further demonstrated that brainstem involvement, hemorrhagic transformation, and elevated cerebrospinal fluid protein are key predictors of poor prognosis, reflecting more extensive central nervous system injury [7]. These findings are particularly relevant to sleep and respiratory physiology, as the brainstem plays a central role in autonomic ventilatory control, especially during sleep.

From a neurophysiological perspective, injury to thalamic and brainstem networks may disrupt the integration between cortical arousal systems and automatic respiratory drive, particularly during sleep, when ventilatory control is predominantly centrally mediated [8]. This disruption may manifest as subclinical or overt central sleep-disordered breathing, including central sleep apnea or sleep-related hypoventilation. Although not systematically described in ANEC, the anatomical distribution of lesions provides a strong rationale for central respiratory dysfunction. Similar mechanisms have been reported in other encephalitic conditions, including autoimmune encephalitis, where central hypoventilation and sleep-related breathing instability may occur despite relatively preserved daytime respiration [8].

Early intervention and resolution of cerebral edema in ANEC have been associated with improved outcomes, suggesting that reversible functional impairment may precede permanent structural injury [7]. In such cases, improvement in respiratory patterns may parallel neurological recovery. However, persistent central apnea or hypoventilation may occur when structural injury, particularly involving the brainstem, is present, which has consistently been identified as a poor prognostic factor in pediatric cohorts [7].

Given the potential involvement of central respiratory control, early evaluation for sleep-related breathing disorders should be considered in children with ANEC, particularly in those with brainstem involvement or otherwise unexplained respiratory instability. Polysomnography may play a key role in identifying these disturbances and guiding timely management.

## Conclusions

ANEC may be associated with severe central sleep apnea as a result of brainstem involvement, representing an underrecognized complication in pediatric patients. This case illustrates that central sleep-disordered breathing in ANEC can be profound yet potentially reversible, with complete resolution following appropriate non-invasive ventilatory support and neurological recovery.

Early recognition and targeted evaluation with polysomnography are essential, particularly in patients with evidence of brainstem dysfunction, as timely intervention may lead to significant improvement and allow safe discontinuation of respiratory support. These findings underscore the importance of including sleep-related breathing disorders within the clinical spectrum of ANEC and support the role of longitudinal sleep monitoring in guiding management and prognosis.
